# Implantable cardioverter defibrillator for primary prevention in patients with non-ischemic cardiomyopathy in the era of novel therapeutic agents- meta-analysis

**DOI:** 10.3389/fcvm.2023.1192101

**Published:** 2023-05-19

**Authors:** Yotam Kolben, Bruria Hirsh Raccah, Ivelin Koev, David Luria, Offer Amir, Yitschak Biton

**Affiliations:** ^1^ Heart Institute, Hadassah Medical Organization and Faculty of Medicine, Hebrew University of Jerusalem, Jerusalem, Israel; ^2^ Department of Cardiovascular Sciences, University of Leicester, Leicester, United Kingdom; ^3^ National Institute for Health and Care Research, Leicester Biomedical Research Centre, Leicester, United Kingdom; ^4^ Heart Research Follow-up Program, University of Rochester Medical Center, Rochester, NY, United States

**Keywords:** ICD (implantable cardioverter-defibrillator), NIDCM, SGLT 2 inhibitors, ARNI, angiotensin receptor neprilysin inhibitor, sudden cardiac death (SCD)

## Abstract

**Background:**

Evidence regarding the mortality benefit of implantable cardioverter defibrillator (ICD) non-ischemic dilated cardiomyopathy (NIDCM) is inconsistent. The most recent randomized study, the DANISH trial, did not find improved outcomes with ICD. However, based on previous studies and meta-analyses, current guidelines still highly recommend ICD implantation in NIDCM patients. The introduction of novel medications for heart failure improved the clinical outcome dramatically. We aimed in this study to evaluate the effect of Angiotensin Receptor-Neprilysin Inhibitors (ARNi) and sodium-glucose transport protein 2 inhibitors (SGLT2i) on the mortality benefit of ICD in NIDCM.

**Methods:**

We used a previous metanalysis algorithm and added an updated comprehensive literature search in PubMed for randomized control trials that examined the mortality benefit of ICD in NIDCM vs. optimal medical treatment. The primary outcome included death from any cause. We did a meta-regression analysis to search for a single independent factor affecting mortality. Using previous data, we evaluated the theoretical effect of ICD implementation on patients treated with SGLT2 inhibitors and ARNi.

**Results:**

No new articles were added to the results of the previous meta-analysis. 2,622 patients with NIDCM from 5 cohort studies published between 2002 and 2016 were included in the analysis. 50% of them underwent ICD implantation for primary prevention of sudden cardiac death, and 50% did not. ICD was associated with a significantly decreased risk for death from any cause compared to control (OR = 0.79, 95%CI: 0.66–0.95, *p* = 0.01, *I*^2^ = 0%). The theoretical addition of ARNi and the SGLT2 inhibitor dapagliflozin did not change the significant mortality effect of ICD (OR = 0.82, 95%CI: 0.7–0.9, *p* = 0.001, *I*^2^ = 0%) and (OR = 0.82, 95%CI: 0.7–0.9, *p* = 0.001, *I*^2^ = 0%). A meta-regression revealed no association between death from any cause and left bundle branch block (LBBB), use of amiodarone, use of angiotensin-converting enzyme inhibitors (ACEi) or angiotensin receptor blockers, year initiated enrollment, and the year ended enrollment (*R*^2^ = 0.0).

**Conclusion:**

In patients with NIDCM, the addition of ARNi and SGLT2i did not affect the mortality advantages of ICD for primary prevention.

**PROSPERO registry number:**

https://www.crd.york.ac.uk/prospero/, identifier: CRD42023403210.

## Introduction

Sudden cardiac death (SCD) is a leading cause of death among patients with dilated cardiomyopathy, accounting for approximately half of the deaths yearly, mostly due to ventricular tachyarrhythmias (1). The potential substrates in these patients include structural abnormalities such as ventricular hypertrophy or scar, metabolic alternations such as intra and extracellular electrolyte and acidity imbalance, inherent electrophysiological changes as action potential and QT prolongation, and neurohormonal dysregulation (2). The data regarding the primary prevention of SCD using implantable cardioverter defibrillator (ICD) is well established in ischemic cardiomyopathy (3–5). These findings correspond with the class 1 recommendation to implant ICD in patients with advanced ischemic cardiomyopathy, in both the European and the American guidelines (6, 7).

However, while SCD is a significant issue in non-ischemic cardiomyopathy (NIDCM), the data regarding its benefit in this population is controversial, unlike the clear benefit in ischemic cardiomyopathy. Naturally, patients with ICD have higher rates of aborted cardiac arrest, but the overall survival benefit is not established. In addition, since the randomized control trials (RCT) which tested the efficacy of ICD for primary prevention were conducted, significant developments in the pharmacological management of heart failure heart failure with reduced ejection fraction (HFrEF) have occurred. Adding angiotensin receptor-neprilysin inhibitors (ARNi) and sodium-glucose sodium cotransporter-2 inhibitors (SGLT2i) to the recommended therapeutic regimen in HFrEF, improved the outcome of patients dramatically, with profound mortality benefit (8). This improvement questions the added benefit of ICD implantation while considering procedural and post-procedural complications.

In this study, we conducted a metanalysis of RCTs to evaluate the efficacy of ICD implantation in NIDCM. We analyzed the data using meta-regression to search for single factors which affected mortality rates, with particular consideration of the year the study was conducted in light of the progression in medical treatments in the past decades. We also made an additional theoretical calculation to assess the benefit of ICD in the era of novel therapeutic agents.

## Methods

### Search strategy

We used the previous metanalysis of Akel et al. (9). We searched for new studies in PubMed until November 27, 2022.

The search strategies incorporate index terms (Mesh) and free text words for the search concepts: nonischemic cardiomyopathy, non-ischemic cardiomyopathy, NIDCM, dilated cardiomyopathy, implantable cardioverter defibrillator, ICD, sudden cardiac death, and cardiac arrest, combined by “AND”, and in each domain, the terms were combined by “OR”. The detailed protocol is documented online in the International Prospective Register of Systematic Reviews registry (PROSPERO registry number: CRD42023403210). Helsinki board approval was waived because this study was a review and meta-analysis.

### Data sources and searches

In the search strategy, we included randomized controlled trials (RCTs). Animal studies, reviews, expert opinions, case reports, case series, duplicated reports, cross-sectional studies, pharmacokinetic studies in healthy adults, editorials, comments, letters to the editor, and studies with a high risk of bias were excluded.

### Study selection and data extraction

Using the Rayyan QCRI web application for systematic review, two investigators (I.K. and Y.K.) independently identified and extracted potential inclusion articles (10). Disagreements were resolved by consensus. The primary outcome included death from any cause. Data were extracted by a single reviewer and subsequently evaluated by the second reviewer.

### Quality assessment and risk of bias risk

Risk of bias and quality was evaluated by the Cochrane Collaboration's Risk of Bias Tool. Assessments of the risk of bias were performed independently by two investigators (11).

### Data synthesis and analysis

Meta-analysis, Meta-regression, and predication interval calculation were performed using Comprehensive Meta-Analysis software. Risk of bias was performed using Review Manager 4.3. Results were summarized using random-effect pooled odds ratios (ORs) with the corresponding 95% confidence intervals (CIs). We used *I*^2^ statistics to assess heterogeneity. Patients in the included studies were not treated with SGLT2i and ARNi. To evaluate the theoretical effect of ICD implementation on patients treated with SGLT2 inhibitors and ARNi, we calculated a hazard ratio (HR) of 0.83 for patients treated by the SGLT2 inhibitors Dapagliflozin and 0.84 for patients treated by ARNi compared to patients treated with conventual therapy (12). We also performed Meta-regression analyses to evaluate whether differences between ICD and non-ICD groups are associated with left bundle branch block (LBBB), use of angiotensin-converting enzyme inhibitor (ACEi) and angiotensin receptor blockers (ARB), year-initiated enrollment, and year ended enrollment.

### Publication bias

Publication bias was planted to be assessed only if the analysis included at least ten studies.

## Results

### Literature search

Citations identified through PubMed search yielded 4,728 citations. Of which, 3 identical duplicates were excluded. No new studies were found. After an abstract assessment, 6 articles were extracted for full-text review. Eventually, five studies were included in the analysis, identical to the search results of the previous metanalysis; the SCD-HeFT trial was divided into two arms (ICD compared to amiodarone and ICD compared to placebo). The meta-analysis included a total of 2,622 patients. Of whom, 1,310 (50%) were treated with ICD, and 1,312 (50%) were not.

### Characteristics of studies

The studies were published between 2002 and 2016. Enrollment years were between 1996 and 2014. There was a total of 2,992 patients. Median follow-up was 24–67.6 months, and the median age was 52–64 years. The median male percentage was 70%–79%. New York Heart Association II–III percentage was 78%–100%, and the median left ventricular ejection fraction was 21.4%–25%. LBBB and right bundle branch block prevalences were 19.6%–82.6% and 3.8%–11.6%, respectively. The prevalence of ACEi/ARB and beta-blockers use was 85.4%–96.7% and 3.85%–91.9%, respectively (Table 1).

**Table 1:** Characteristics of studies included.

Study	CAT	AMIOVIRT	DEFINITE	SCD-HeFT	DANISH
Author	Bänsch et al. (13)	Strickberger et al. (14)	Kadish et al. (15)	Bardy et al. (16)	Køber et al. (17)
Journal	Circulation	JACC	NEJM	NEJM	NEJM
Year published	2002	2003	2004	2005	2016
Enrollment years	1991–1997	1996–2000	1998–2002	1997–2001	2008–2014
Patients	104	103	458	841	1,116
Comparison	ICD vs. OMT	ICD vs. Amiodarone vs. OMT	ICD vs. OMT	ICD vs. Amiodarone vs. OMT	ICD vs. OMT
Inclusion criteria	Age 18–70	Age >18	LVEF ≤ 35%	Age >18	Non-ischemic HF
	Symptomatic DCM for <9 months	LVEF < 35%	Symptomatic HF	NYHA II–III	LVEF ≤ 35%
	LVEF < 30%	Asymptomatic NSVT	Non-ischemic DCM	Chronic stable HF	NYHA II or III
	NYHA II–III	NYHA I–III	Ambient arrythmia	LVEF < 35%	NYHA IV if CRT is planned
					NT-PROBNP > 200 pg per ml
Median follow-up (months)	66	24	29	45.5	67.6
Median age (years)	52	59	58.3	60	64
Male (%)	79	70	71	77	73
NYHA II–III (%)	100	84	78	100	99
LVEF (%)	24	22	21.4	25	25
Atrial fibrillation (%)	15.3	NA	24.4	NA	22.2
LBBB (%)	82.6	47.5	19.6	NA	53.5
RBBB (%)	3.8	11.6	3.2	NA	3
Mean QRS (ms)	108	NA	115	NA	145
ACEi + ARB (%)	96.2	85.4	96.7	NA	96.5
Beta-blockers (%)	3.8	51.5	84.9	NA	91.9
Diuretics (%)	86.5	68.9	86.7	NA	NA
Amiodarone (%)	NA	49.5	5.2	NA	5.9
Digoxin (%)	80.8	68.9	41.9	NA	NA
Nitrate (%)	28.8	NA	11.1	NA	NA
MCA (%)	NA	NA	NA	NA	57.9
CCB (%)	11.5	NA	NA	NA	NA

ACEi, angiotensin converting enzyme inhibitor; ARB, angiotensin receptor blocker; CCB, calcium channel blocker; CRT, cardiac resynchronization therapy; DCM, dilated cardiomyopathy; HF, heart failure; ICD, implantable cardioverter defibrillator; LBBB, left bundle branch block; LVEF, left ventricular ejection fraction; MCA, mineralocorticoid receptor antagonist; NYHA, New York heart association; OMT, optimal medical treatment; RBBB, right bundle branch block.

### Quality assessment

The overall risk of bias of three randomized controlled studies evaluated by the Cochrane Collaboration's Risk of Bias Tool was low. The risk of bias assessment is summarized in Supplementary Figure S2.

We could not test for funnel plot asymmetry to assess possible publication bias because less than ten studies were included in the meta-analysis (18).

### Death from any cause

**Figure 1:**
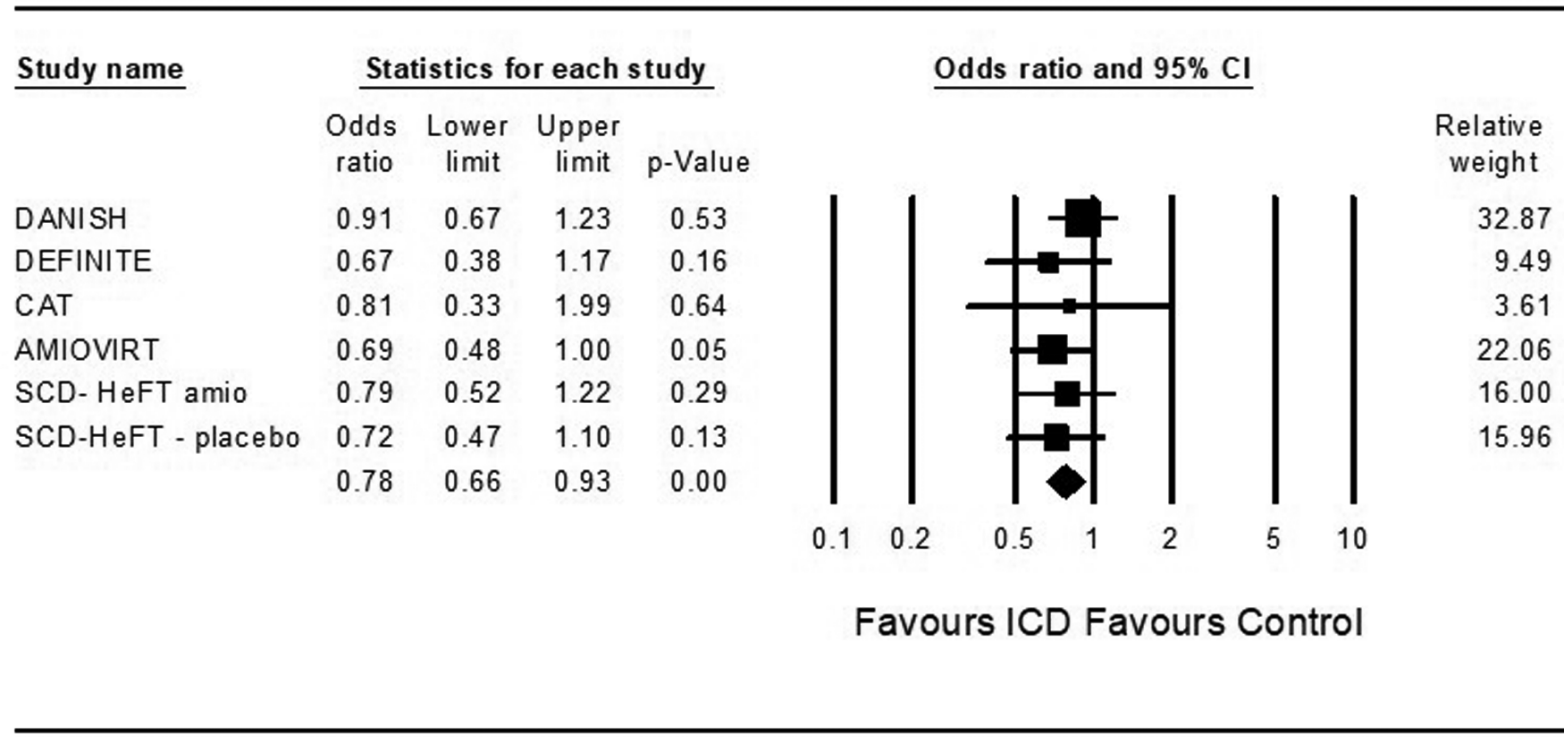
Fidings from 5 randomized controlled trial of implantable cardioverter defibrillator vs. medical therapy in non-ischemic cardiomyopathy.

Five studies examined death from any cause among ICD patients. ICD was associated with a significantly decreased risk for death from any cause compared to control (OR = 0.79, 95%CI: 0.66–0.95, *p* = 0.01, *I*^2 ^= 0%) (Figure 1).

**Figure 2:**
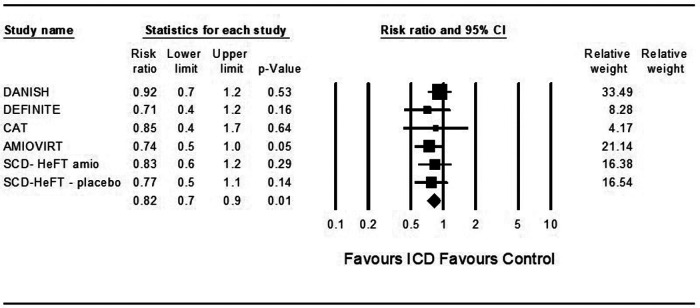
Results of the trials with the theoretic addition of angiotensin receptor-neprilysin inhibitor to all patients.

**Figure 3:**
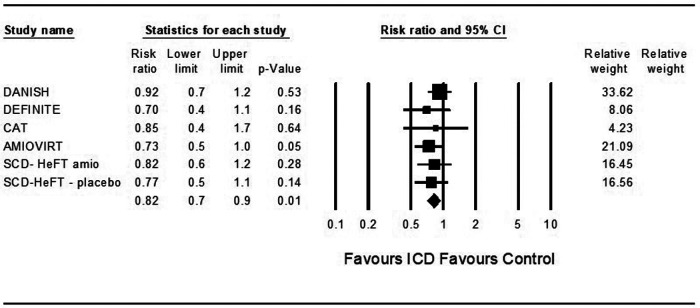
Results of the trials with the theoretic addition of dapagliflozin to all patients.

In theoretical analysis, ICD was associated with a significantly decreased risk for death from any cause compared to control in patients treated with ARNi and the SGLT2 inhibitor dapagliflozin (OR = 0.82, 95%CI: 0.7–0.9, *p* = 0.001, *I*^2 ^= 0%) and (OR = 0.82, 95%CI: 0.7–0.9, *p* = 0.001, *I*^2 ^= 0%) (Figures 2, 3).

The prediction interval reflects the dispersion in true effects. Since tau-squared is estimated as zero, we assume that all studies share a common effect size and there is no dispersion of true effects. Therefore, we do not report a prediction interval.

### Meta-regression

A meta-regression revealed no association between death from any cause and age, LBBB, use of amiodarone, use of ACEi or ARBs, year initiated enrollment, and the year ended enrollment (*R*^2 ^= 0.0) (Supplementary Figures S3–S7).

## Discussion

SCD is a significant issue in NIDCM. Nevertheless, the mortality benefit of ICD in these patients is controversial. The Danish Study to Assess the Efficacy of ICDs in Patients with Non-ischemic Systolic Heart Failure on Mortality (DANISH) (17), the largest and most recent RCT, did not show a significant reduction in all-cause mortality, challenging common practice. The negative results of this trial may be attributed to improvements in therapy in HF patients. Medical treatment in most of the studies is outdated, and remarkable advancements have been made since (8). In the PARADIGM-HF, ARNi reduced all-cause mortality and cardiovascular death, specifically reducing sudden death (HR 0.8, *p* = 0.008) (19). Patients with HFrEF and after ICD implantation had fewer events of ventricular tachyarrhythmias after ARNi treatment initiation (20). SGLT2i improved cardiovascular outcomes in HFrEF patients (12, 21), but it was not proven to affect ventricular arrhythmias (22, 23).

In this study, we rely on previous metanalysis (9), which suggested that ICD reduces all-cause mortality in patients with NIDCM based on RCT data. A notable study included in the analysis, the DANISH trial, may have weakened the results but did not alter its significance. One large study which was included in other meta-analyses, the Comparison of Medical Therapy, Pacing and Defibrillation in Heart Failure (COMPANION) trial (24), was excluded in this analysis because the subgroup analysis of NIDCM was reported only for Cardiac resynchronization therapy (CRT) with defibrillator vs. optimal medical treatment without CRT, neglecting the effect of CRT on mortality. Previous metanalysis showed similar results as ours (25, 26), giving weight to the class 1 and class IIa recommendation to implant an ICD as primary prevention in NIDCM patients in the American and European guidelines, respectively (6, 7).

Using meta-regression, we examined the effect of a single attribute on the results, and no single factor was identified to affect mortality significantly. Most studies were conducted over two decades ago, so we assumed enrollment years would affect the results. Still, most studies were done in parallel, which may affect our analysis. In a subgroup analysis of the DANISH trial, a mortality benefit was found in patients under 60 in implementing an ICD (17). In a long-term follow-up of the DANISH trial, ICD had a mortality benefit in patients under 70 years of age (27). These data suggest the logical understanding that younger patients will enjoy ICD implantation more than older patients. However, in our meta-regression, the median age was not found to be a significantly affecting factor. In the effort to predict which HF patients will benefit from ICD implantation, previous data demonstrated that scar mass as appeared in Magnetic Resonance Imaging may be the key (28). However, while the DANISH-MRI substudy showed that late gadolinium enhancement predicted all-cause-mortality, it did not identify the patients who will benefit from receiving and ICD (29).

Since the DANISH trial was published, novel therapeutic agents were introduced and significantly impacted outcomes. We assumed that in the theoretical addition of ARNi and SGLT2i to the analysis, ICD would not show significant mortality benefit. However, the addition of these medications did not alter the results, strengthening the robustness of ICD in NIDCM patients. Controlled trials are still needed in this new therapeutic era.

We used in this metanalysis only RCTs, which strengthened the results. In addition, the prediction interval was significant, and the heterogenicity was low, supporting current guidelines.

There are a few limitations to this study. First, all the trials were not utterly blinded as no sham procedures were performed. Second, as ischemic cardiomyopathy is more common than NIDCM, we cannot apply our results to this group of patients. Furthermore, NIDCM patients suffer from coronary microvascular dysfunction, suggesting combined etiology for some patients (30). Third, most studies are outdated regarding medical treatments, affecting the initial metanalysis results.

## Conclusion

Implantation of ICD as primary prevention in advanced NIDCM has a mortality benefit. Most of the trials which assessed its efficacy were initiated in the previous millennium. The theoretical addition of ARNi and SGLT2i to the patients did not alter the result, supporting further use of ICD for primary prevention in NIDCM.

## Data Availability

The raw data supporting the conclusions of this article will be made available by the authors, without undue reservation.
